# Congenital Transmission of *Trypanosoma cruzi* Infection in Argentina

**DOI:** 10.3201/eid0901.020274

**Published:** 2003-01

**Authors:** Ricardo E. Gürtler, Elsa L. Segura, Joel E. Cohen

**Affiliations:** * Departamento de Ecología, Genética y Evolución, Universidad de Buenos Aires, Buenos Aires, Argentina; † Centro Nacional de Diagnóstico e Investigación en Endemo-epidemias, Buenos Aires, Argentina; ‡ Laboratory of Populations, Rockefeller and Columbia Universities, New York, New York USA

**Keywords:** congenital, Chagas disease, transmission, trypanosomiasis, control, surveillance, fertility, demography, research

## Abstract

*Trypanosoma cruzi*, the causative agent of Chagas disease, infects 10–18 million people and may be transmitted to the newborn. Using various data sources, we estimated that nearly 850 congenital cases occurred in Argentina in 1993, or 6.3 expected cases per each reported case in 1994 and in 1994–2001. The congenital transmission of *T. cruzi* constitutes a sizeable public health problem in the region.

Congenital cases of *T. cruzi* are mostly asymptomatic or monosymptomatic and seriously affect the newborn’s survival and illness rate (*2*,*3*). Such cases cannot be prevented because the available drugs have adverse effects, but early detection and prompt treatment are frequently successful (*3*). However, as screening of pregnant women and newborns has not been routinely conducted in most *T. cruzi*–endemic countries, the magnitude of the congenital transmission of this pathogen as a public health problem has not been established. Having such an estimate would be important for making policy recommendations and health service planning. Our study estimates the annual number of congenital Chagas cases that occurred in Argentina recently and compares them with official case reports.

## Materials and Methods

For a given province, year, maternal age group, and the estimated number of live patients with congenital *T. cruzi* infection were computed as the total number of live newborns (*f*), times the probability of a woman’s being infected with *T. cruzi* (*p*), times the probability of transmitting *T. cruzi* to the live newborn (*t*). Province and age-specific numbers of live births and maternal seroprevalence rates of infection are needed to provide a countrywide annual estimate.

The total number of live births in 1991 (694,776 newborns), according to the mother’s age group and province (*4*), changed very little during the 1990s. We categorized births uniformly within each maternal 5-year age class because data on births of single-year age classes were not available; we did not count the very small number of births in other countries or with unspecified birthplace.

The probability of a woman’s being infected with *T. cruzi* varies with age; however, it may be estimated from the seroprevalence for *T. cruzi* in men of the same age group because no evidence of a gender-related excess risk of infection has been detected (*5*,*6*). For Argentina, the available province-specific seroprevalence rates of *T. cruzi* infection for young men drafted into military service in 1965–1969 (when they were 21 years of age) and annually from 1981 to 1993 (when they were 18 years) showed quite different temporal trends (*7*). Each recruit age class was a randomly selected birth cohort; unhealthy persons were excluded after blood samples were taken. To reconstruct maternal seroprevalence rates in 1993, we assumed a closed, steady-state population between year of diagnosis and 1993. This assumption meant that a) the fraction infected in each birth cohort remained stable because most *T. cruzi* infections were acquired during childhood, and specific treatment of infected adults was uncommon except for male recruits and legal immigrants; and b) men and women ages 15–50 had similar age-specific death and emigration rates because most findings of *T. cruzi–*specific pathology in women occur at postreproductive ages (*8*). Potential differences in cohort-specific rates of recruitment or loss of infected women through differential migration associated with *T. cruzi* infection were ignored, although internal migrations may modify the expected number of cases, depending on the interplay between fertility, *T. cruzi* infection, age at migration, and source and destination of migrants. We ignored the contribution of increasing numbers of adult immigrants from neighboring *T. cruzi–*endemic countries since the 1950s. We tentatively assumed that the chance of being pregnant and having an uneventful pregnancy was not affected by infection with *T. cruzi*, but present evidence is controversial.

The province-specific maternal prevalence of *T. cruzi* infection in 1993 comprised the partial contributions of mothers who were 18 years old in 1993 (born in 1975) and to whom we assigned the seroprevalence rate of 18-year-old men assessed in 1993, and so on through mothers ages 30 years in 1993 (born in 1963) and to whom we assigned the seroprevalence rate of 18-year-old men assessed in 1981. For mothers >30 years of age in 1993, we assigned the rate from 1965–1969 data to 1967 and assumed that the prevalence followed a linear trend between 1967 and 1981. The few 14- to 17-year-old mothers were assigned the 18-year-olds’ seroprevalence assessed in 1993. Tierra de Fuego was excluded from calculations because the time series had numerous missing data. Calculations were carried out in an Excel spreadsheet, available on request.

The probability of congenital transmission from pregnant women seropositive for *T. cruzi* has been extremely variable (range 0.005–0.117) among countries and geographic areas (*1*–*3*), and its determinants are little known (*9*,*10*). From the latest review (*3*), we estimated the median *t* as 0.025 (interquartile range 0.02–0.04). We assumed that *t* was not modified by the mother’s age (*9*,*10*) because most mothers were in the indeterminate or chronic phase of infection in which the parasitemia levels are low and age independent. We ignored potential geographic variations and parasite strain effects on *t*.

## Results

**Figure 1:**
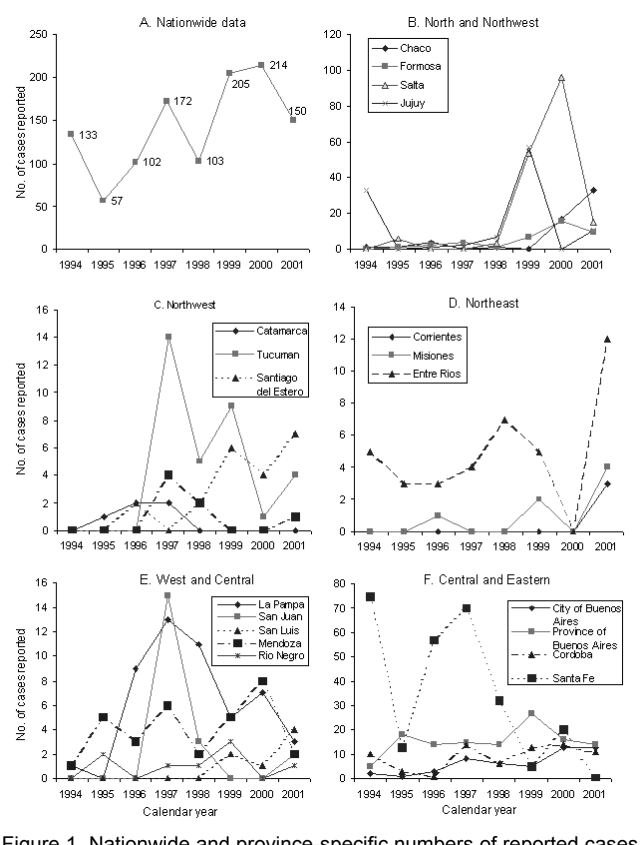
Nationwide and province-specific numbers of reported cases of congenital *Trypanosoma cruzi* infection notified to the Ministry of Health of Argentina. Mean coefficient of variation among provinces over time, 266%; range 39% to 283%. For the city and Province of Buenos Aires, we used the 1994–2001 data corrected by the Chagas National Surveillance System.

The National System of Epidemiological Surveillance reported (*11*) a total of 1,080 congenital cases of *T. cruzi* in 1994–2001 (annual mean 135; standard deviation 35), with no significant time trend and very large asynchronic variations among and within provinces (Figure 1). As demonstrated for leishmaniasis surveillance in Argentina (*12*), inconsistencies among provincial, national, and Chagas surveillance reports of congenital cases were frequent and led to substantial underreporting.

**Figure 2:**
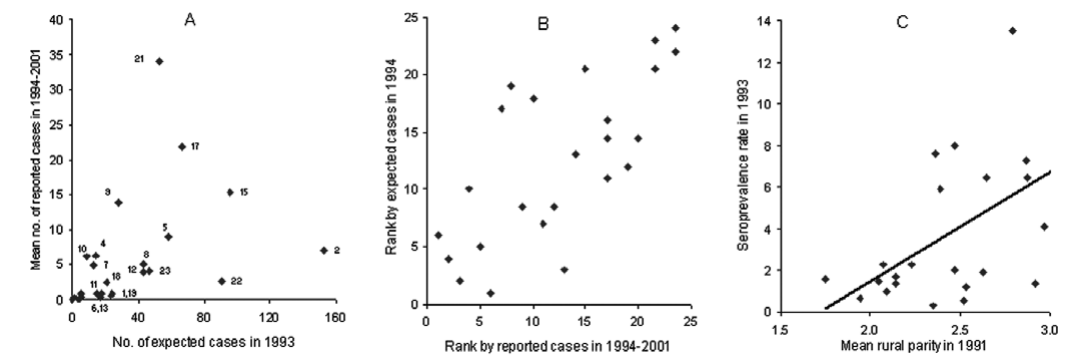
(A) Numbers of expected (in 1993) and reported (mean of 1994–2001) cases of congenital *Trypanosoma cruzi* infection. (B) Ranked ordering of provinces according to numbers of expected (in 1993) and total reported cases (1994–2001) of congenital *T. cruzi* infection; Spearman’s correlation coefficient (R) = 0.711, n=24, p<0.0001. 1, Catamarca; 2, Chaco; 3, Chubut; 4, City of Buenos Aires; 5, Cordoba; 6, Corrientes; 7, Entre Rios; 8, Formosa; 9, Jujuy; 10, La Pampa; 11, La Rioja; 12, Mendoza; 13, Misiones; 14, Neuquen; 15, Province of Buenos Aires; 16, Rio Negro; 17, Salta; 18, San Juan; 19, San Luis; 20, Santa Cruz; 21, Santa Fe; 22, Santiago del Estero; 23, Tucuman. (C) Relationship between seroprevalence rates of *T. cruzi* infection in military recruits in 1993 and mean rural parity in 1991 (R=0.541, n=23, p<0.01). The City of Buenos Aires, which does not have a rural area, was excluded from analysis.

A total of 846 congenital cases were estimated for 1993 (Figure 2A). An example of the calculations for the province of Buenos Aires is given in the Appendix. The expected annual number of congenital cases peaked in Chaco (153 cases) and the province of Buenos Aires (96 cases); the latter had rare domiciliary triatomine infestations and a large number of immigrants from Chagas-endemic provinces during 1947 to the 1970s. Santiago del Estero, with high seroprevalence and fertility rates but very low human population, ranked 3rd (90 cases). The ratio between our conservative estimate of congenital cases in 1993 (846 cases) and official notifications in 1994 and 1994–2001 (135 cases) was 6.3: 1. A very rough calculation that used countrywide averages (*f* = 687,051; *p* = 0.019; *t* = 0.025) yielded 326 congenital cases, or 38% of the above estimate.

Extreme differences between observed and reported mean numbers of congenital cases occurred in Chaco and Santiago del Estero, followed by Formosa, Tucuman, and Mendoza (Figure 2A), suggesting strong underreporting. Santa Fe, Jujuy, Salta, and the province of Buenos Aires reported most cases. The ranked province-specific total numbers of cases estimated for 1993 and reported officially from 1994 to 2001 were significantly correlated (Figure 2B) and thus provided a qualitative hierarchic ordering of provinces in terms of the likely burden of congenital cases.

Additional calculations tend to support our rough estimates. A pilot control program in a public maternity facility, where 37.8% of all births in Tucuman took place, detected 32 congenital cases over 28 months (mean 13.7 cases per year) (*13*). If we assume this was a random sample, the annual number of congenital cases projected to the whole province was 36.2 cases, which is roughly close to the expected number of 46.7 cases.

Schmuñis (*1*) estimated that 1,593 congenital cases of *T. cruzi* occurred annually in Argentina around 1985, on the assumptions that the maternal seroprevalence equaled the overall seroprevalence of *T. cruzi* in blood banks (6.96%); that *t* = 0.03; and that no age-specific variations in fertility and prevalence of infection occurred. When we used data on the seroprevalence of *T. cruzi* among 131,909 pregnant women (4.4%) from 15 Argentine provinces in 2000, and among 153,266 women (5.7%) from 20 provinces in 2001 (Sonia Blanco, unpub. data), where *f* = 700,000 and *t* = 0.025, a similar calculation yields 770 and 997 estimated congenital cases for 2000 and 2001, respectively. However, because mean rural parity in 1991 was positively and significantly associated with *T. cruzi* seroprevalence in military recruits in 1993 at a province level (Figure 2C), fertility and maternal infection also may be positively associated at the individual level in rural settings. Therefore, the use of average, province-wide fertility rates would underestimate both the number of newborns from infected women and the occurrence of congenital cases.

DiscussionThe congenital transmission of *T. cruzi* appears to be a sizeable public health problem in Argentina, where it has already surpassed the number of vector-mediated acute cases by a factor of 10, and probably elsewhere in the region. Despite a long-term decreasing trend in the human prevalence of *T. cruzi*, for which we need increased prevention measures, infected women of reproductive age will still give birth in the foreseeable future. Available data favor a short-term policy of antenatal diagnosis of pregnant women for *T. cruzi* infection and follow-up of their newborns. Increased international migrations from *T. cruzi*–endemic Latin American countries suggest the need for an increased awareness among obstetricians, neonatologists and pediatricians. Effective, nontoxic drugs that may be administered to prospective mothers or pregnant women to reduce the likelihood of congenital transmission are clearly needed.

## Supplementary Material

Appendix Example of the calculations involved in the estimate of the number of congenital cases of Trypanosoma cruzi infection for the Province of Buenos Aires, 1993
