# Supplementary material for: Congenital Transmission of Trypanosoma cruzi Infection in Argentina
Source: Emerg Infect Dis. 2003 Jan;9(1):29–32. doi: 10.3201/eid0901.020274 (PMC2873757; doi:10.3201/eid0901.020274)
Supplement: Appendix — Example of the calculations involved in the estimate of the number of congenital cases of Trypanosoma cruzi infection for the Province of Buenos Aires, 1993 [file 02-0274_app-s1.pdf]

Publisher: CDC; Journal: Emerging Infectious Diseases
Article Type: Research; Volume: 9; Issue: 1; Year: 2003; Article ID: 02-0274
DOI: 10.3201/eid0901.020274 ; TOC Head: Research

Appendix. Example of the calculations involved in the estimate of the number of congenital cases of *Trypanosoma cruzi* infection for the Province of Buenos Aires, 1993

Mother's birth year	Seroprevalence of *T. cruzi*	No. of live newborns	No. of live newborns from infected women	No. of expected congenital cases
1946	4.70	281	13.22	0.3
1947	4.57	281	12.85	0.3
1948	4.44	281	12.49	0.3
1949	4.31	1,322	56.99	1.4
1950	4.18	1,322	55.28	1.4
1951	4.05	1,322	53.57	1.3
1952	3.92	1,322	51.86	1.3
1953	3.79	1,322	50.15	1.3
1954	3.66	4,623	169.42	4.2
1955	3.54	4,623	163.44	4.1
1956	3.41	4,623	157.46	3.9
1957	3.28	4,623	151.47	3.8
1958	3.15	4,623	145.49	3.6
1959	3.02	8,628	260.37	6.5
1960	2.89	8,628	249.20	6.2
1961	2.76	8,628	238.04	6.0
1962	2.63	8,628	226.87	5.7
1963	2.50	8,628	215.70	5.4
1964	2.39	12,703	303.61	7.6
1965	2.31	12,703	293.44	7.3
1966	0.18	12,703	22.87	0.6
1967	0.15	12,703	19.05	0.5
1968	1.19	12,703	151.17	3.8
1969	1.04	12,571	130.74	3.3
1970	1.08	12,571	135.77	3.4
1971	0.77	12,571	96.80	2.4
1972	0.68	12,571	85.48	2.1
1973	0.65	12,571	81.71	2.0
1974	0.71	6,160	43.74	1.1
1975	0.70	6,160	43.12	1.1
1976	0.70	6,160	43.12	1.1
1977	0.70	6,160	43.12	1.1
1978	0.70	6,160	43.12	1.1
1979	0.70	695	4.86	0.1

Publisher: CDC; Journal: Emerging Infectious Diseases
Article Type: Research; Volume: 9; Issue: 1; Year: 2003; Article ID: 02-0274
DOI: 10.3201/eid0901.020274 ; TOC Head: Research

Total				95.6
